# A new species of the genus *Petrolisthes* Stimpson (Crustacea, Decapoda, Porcellanidae) from the Central Pacific, with remarks and new records for *P. aegyptiacus* Werding & Hiller

**DOI:** 10.3897/zookeys.617.9893

**Published:** 2016-09-15

**Authors:** Alexandra Hiller, Bernd Werding

**Affiliations:** 1 Smithsonian Tropical Research Institute, Apartado 0843–03092, Panama, Republic of Panama; 2 Institut für Tierökologie und Spezielle Zoologie der Justus-Liebig-Universität Giessen, Heinrich-Buff-Ring 29 (Tierhaus), D-35392 Giessen, Germany

**Keywords:** Crustacea, Porcellanidae, Petrolisthes, new species, Indo-West Pacific, species complex, range extension

## Abstract

*Petrolisthes paulayi* **sp. n.** is described from specimens collected in French Polynesia. The new species belongs to an assemblage of morphologically similar Indo-West Pacific (IWP) species, here designated as the “mesobranchial-spine group”. All species in the group bear carapace spines, including one or more mesobranchial spines, and transverse, piliferous striations on the dorsal surface of carapace and chelipeds. *Petrolisthes paulayi* sp. n. is distinguishable from all species in the group by its forwardly produced, trilobate front, and a characteristic combination of carapace spines. We also report on the range extension and live coloration of *Petrolisthes aegyptiacus* Werding & Hiller, another species of the mesobranchial-spine group, so far considered a Red Sea endemic. Specimens from the Mascaréne Islands confirm that the geographic range of the species extends to the southern Indian Ocean. While specimens morphologically similar to *Petrolisthes aegyptiacus*, and collected in the Line and Society Islands, suggest a large range extension to the Central Pacific, it is probable that these individuals represent an undescribed species closely related to *Petrolisthes aegyptiacus*.

Indo-West Pacific

## Introduction

The genus *Petrolisthes* Stimpson, 1858, is the most species-rich genus of the family Porcellanidae (Crustacea, Decapoda, Anomura, Galatheoidea), with 109 species worldwide (Dong et al. 2010; Hiller and Werding 2010; Osawa and McLaughlin 2010; Osawa and Maenosono 2011; Osawa and Uyeno 2013; Naderloo and Apel 2014; Werding and Hiller 2015). All species of the genus share a flattened body shape, but they are morphologically highly diverse, and were therefore classified by Haig (1960) into five “natural divisions”. The largest and worldwide distributed division is characterized by teeth and spines on carapace and pereopods, and contains Ortmann’s (1897) *Petrolisthes galathinus*-group (surface of carapace and pereopods with marked transverse, pilliferous striations) and *Petrolisthes lamarckii*-group (surface of carapace and pereopods relatively smooth). Kropp (1984) upgraded this group with newly described species. The two groups are taxonomically difficult because they contain numerous complexes of species with high intraspecific variation in diagnostic characters that sometimes overlap interspecifically. Some of these complexes have been disentangled by examining large series of specimens from different localities (e.g. Kropp 1984; Werding and Hiller 2015; Osawa and Ng 2016), and by comparing DNA sequences from different conspecific and sympatric morphotypes suspected to represent different species (Hiller et al. 2006; Hiller and Werding 2007, 2010).

While all Atlantic and East Pacific (EP) species (with one exception, *Petrolisthes sanfelipensis* Glassell, 1936) of the “*Petrolisthes galathinus* group” do not bear spines on the mesobranchial margins of the carapace, a group of morphologically similar Indo-West Pacific (IWP) species is characterized by a set of one or more such spines. This group was represented until the end of the 1970s by *Petrolisthes scabriculus* (Dana, 1852) and *Petrolisthes militaris* (Heller, 1862) only. In the last three decades, six morphologically similar species were described: *Petrolisthes celebesensis* Haig, 1981, *Petrolisthes perdecorus* Haig, 1981, *Petrolisthes heterochrous* Kropp, 1986, *Petrolisthes nanshensis* Yang, 1996, *Petrolisthes aegyptiacus* Werding & Hiller, 2007, and *Petrolisthes holthuisi* Hiller & Werding, 2010. Only *Petrolisthes aegyptiacus* is restricted to the Indian Ocean, and has been so far reported as the only endemic porcellanid from the Red Sea (Werding and Hiller 2007). We designate this assemblage of species as the mesobranchial-spine group. Here, we describe a new species of this group, *Petrolisthes paulayi* sp. n., from specimens recently collected in the Central Pacific by the Florida Museum of Natural History. We also provide a comprehensive and summarized table with the combination of morphological characters leading to the identification of all species of the group, with minimal ambiguity. Additionally, we discuss new information on the geographic range and coloration of *Petrolisthes aegyptiacus*.

## Material and methods

Specimens deposited in the collections of the Florida Museum of Natural History, University of Florida
(UF), Gainesville, U.S.A., the Muséum National d’Histoire Naturelle (MNHN), Paris, France, and the Justus-Liebig University
(JLU), Giessen, Germany, were examined. Individuals of the new species and of *Petrolisthes aegyptiacus* were counted and sexed, and carapace length and width of the holotype and largest paratype male and female were measured in mm using a stereoscope with a micrometer. Table 1 lists morphological diagnostic characters useful to identify all nominal species of the IWP mesobranchial-spine group, including *Petrolisthes paulayi* sp. n.

**Table 1.** Diagnostic characters for identification of the *Petrolisthes* species comprising the Indo-West Pacific mesobranchial-spine group. Diagnostic characters for identification of the *Petrolisthes* species comprising the Indo-West Pacific mesobranchial-spine group. SOS = number of supraocular spines; EBS = number of epibranchial spines; MBS = number of mesobranchial spines; CTF = conspicuous trilobate front; SP-WL1 = presence of spur-like spine on walking leg 1; LSC = abundant, long setae on carapace.

Species	SOS	EBS	MBS	CTF	SP-WL1	LSC
*Petrolisthes aegyptiacus*	1	1	2	No	No	No
*Petrolisthes celebesensis*	0	1	1	No	No	No
*Petrolisthes heterochrous*	1	2	1-2	No	No	No
*Petrolisthes holthuisi*	1	2	2	No	Yes	No
*Petrolisthes militaris*	1	2	>2	No	No	No
*Petrolisthes nanshensis*	0	2	1	No	No	No
*Petrolisthes paulayi* sp. n.	1-2	2	2	Yes	No	No
*Petrolisthes perdecorus*	1	2	2-3	Yes	No	Yes
*Petrolisthes scabriculus*	2	2	>2	No	No	No

## Results

### Systematic account Family Porcellanidae

#### Petrolisthes paulayi sp. n.

Taxon classificationAnimaliaDecapodaPorcellanidae

http://zoobank.org/505F537B-319C-460F-9C53-47BF862FE799

Figs 1 , 2 , 3

##### Material.

Holotype: UF43955, male, Line Islands, Palmyra Atoll, N side of Atoll, outer reef slope, from dead Pocillopora cf. verrucosa head, 10.6 m.

Paratypes: UF43956, 1 male, same collection data as holotype; UF10692, 1 male (with bopyrid), 1 ovigerous female, Kiribati, Line Islands, Tabuaeran Atoll, SSW side of Atoll, outer reef slope, under rock, 10-23 m; UF10693, 2 females (1 ovigerous), Kiribati, Line Islands, Tabuaeran Atoll, outer reef slope, from *Halimeda* sample, 10-23 m; UF10711, 1 female, Line Islands, Tabuaeran Atoll, W side, S of Main Reef Pass, outer reef slope, from dead Pocillopora cf. verrucosa head, 10-15 m; UF15894, 1 male, (photographed specimen, Fig. 3), French Polynesia, Society Islands, Moorea, Haapiti, just NW of Matauvau Pass, outer reef slope, 15-23 m.

**Figure 1.**
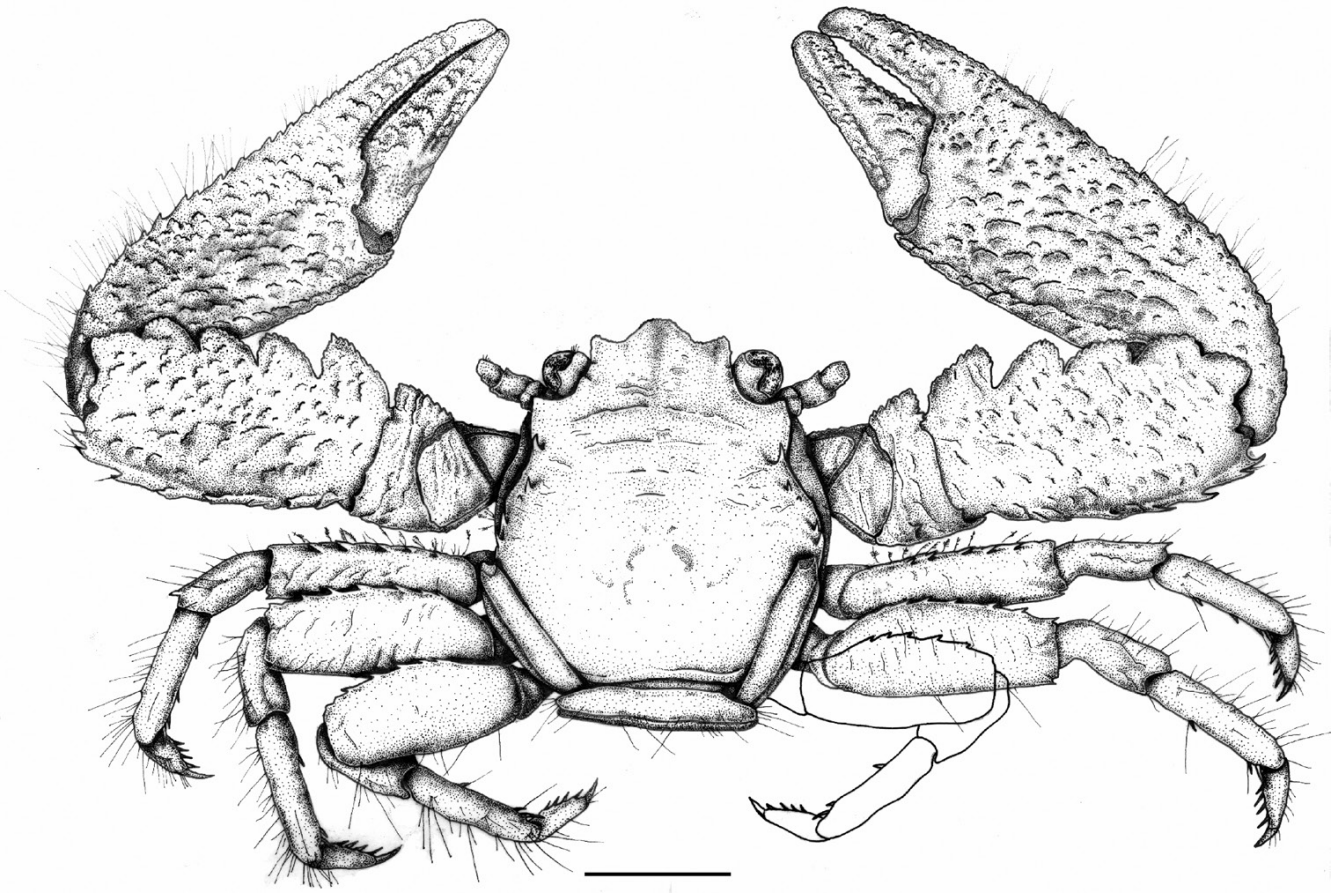
*Petrolisthes paulayi* sp. n. Male, holotype, dorsal view, UF43955, Line Islands, Palmyra Atoll. Right third walking leg supplemented. Scale bar: 2 mm.

**Figure 2.**
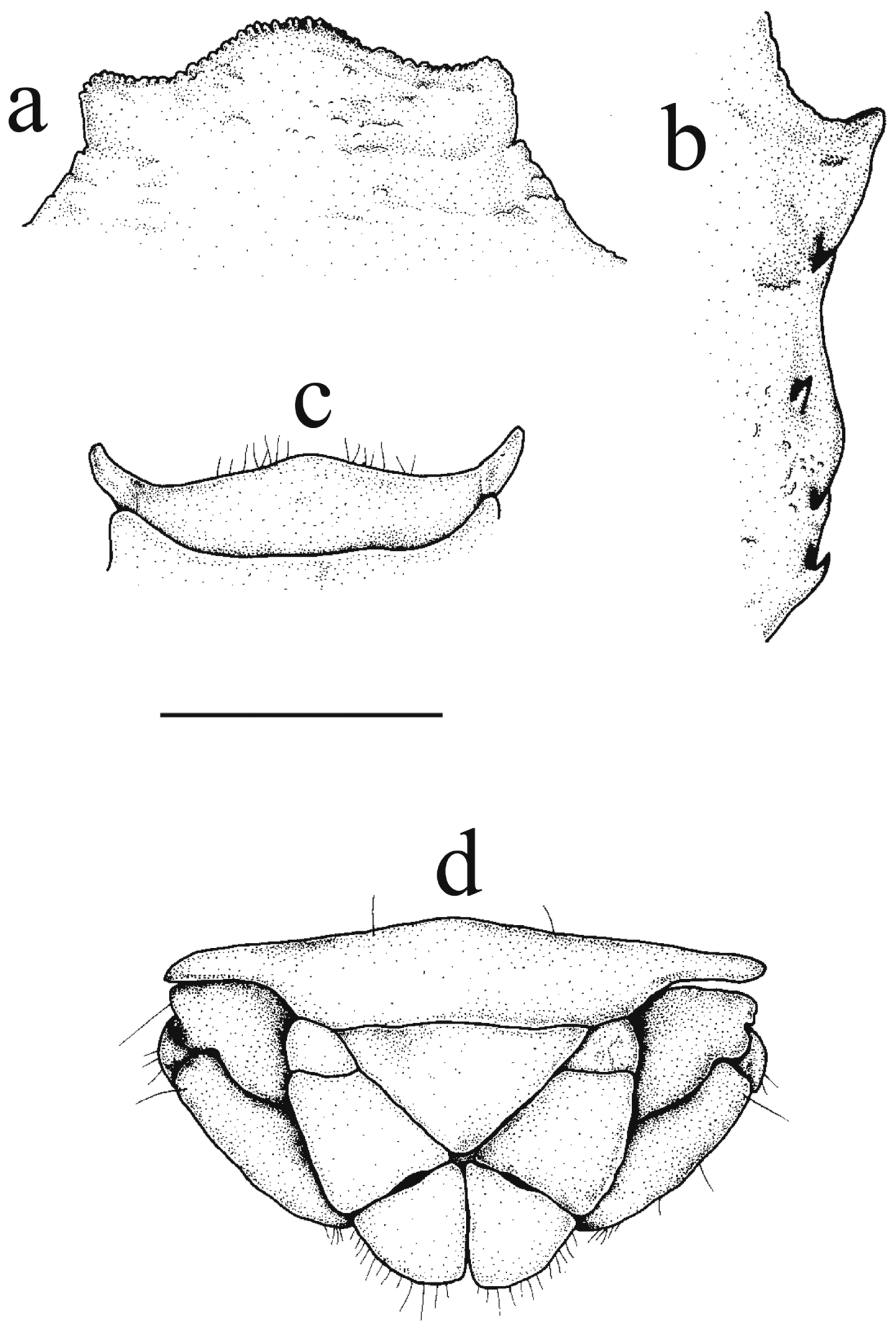
*Petrolisthes paulayi* sp. n. Male, holotype, UF43955, Line Islands, Palmyra Atoll. **a** Carapace front, dorsal view **b** right, lateral margin of carapace showing epibranchial and mesobranchial spines, dorsal view **c** third thoracic sternite, ventral view **d** last abdominal segment, telson and uropods, external view. Scale bar: 1 mm.

**Figure 3.**
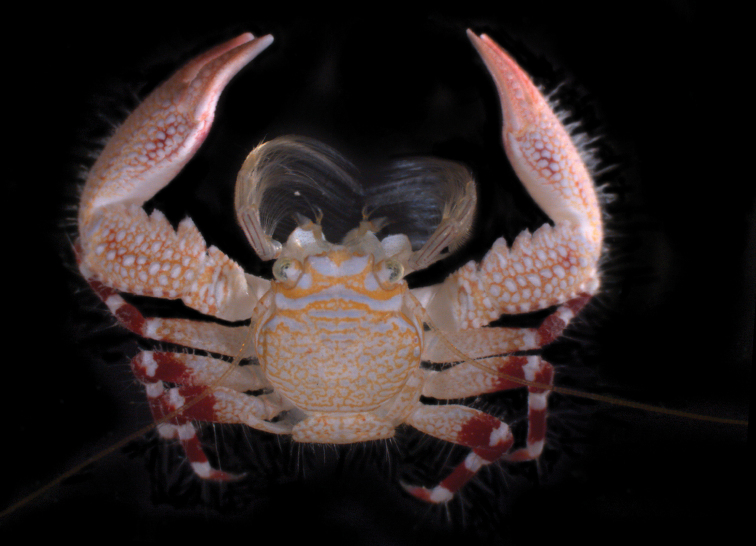
*Petrolisthes paulayi* sp. n., UF15894, Society Islands, Moorea. Photographed by the Moorea Biocode Marine Invertebrate Team (catalogue number dMBC08_03896, Florida Museum of Natural History).

##### Other material.

UF10588, 12 specimens, Line Islands, Palmyra Atoll, Line, N side of Atoll, outer reef slope, dead Pocillopora cf. verrucosa head, 10.6 m; UF41926, 1 male, Kiribati, Line Islands, Starbuck, Starbuck Island, dead *Pocillopora*, 12 m; UF41916, 1 female, Kiribati, Line Islands, Starbuck, Starbuck Island, 12 m; UF40590, 2 males, 1 ovigerous female, Kiribati, Line Islands, Starbuck, Starbuck Island, 7 m; UF41980, 4 males, 4 females (3 ovigerous), Kiribati, Line Islands, Vostok, Vostok Island, dead *Pocillopora*, 10 m.

##### Measurements.

Male holotype: carapace length 4.5 mm; carapace width 4.2 mm.

Largest male paratype: carapace length 4.8 mm; carapace width 4.5 mm.

Largest female paratype: carapace length 4.5 mm; carapace width 4.3 mm.

##### Description.

Carapace (Figs 1, 3) slightly longer than broad. Front (Fig. 2a) trilobate, markedly produced beyond eyes, median lobe broadest, rounded, overreaching the slender, forwardly produced lateral lobes; frontal borders finely serrated. Orbits deeply rounded, inner margin armed with a small supraorbital spine, followed mostly by a second tubercle-like elevation that is sometimes armed with a spine; infraorbital angles forming an acute edge. Lateral margins (Fig. 2b) with a prominent epibranchial spine on epibranchial edge, and followed by a second smaller spine; two strong spines on mesobranchial margin. Carapace with few transverse, piliferous striations on protogastric ridge and on gastric region; epibranchial region rugose.

Third thoracic sternite (Fig. 2c) anteriorly trilobate, median lobe broad, lateral lobes slender, exceeding median lobe.

Telson (Fig. 2d) with seven plates.

First movable segment of antenna with an anterodistal slender projection bearing a narrow tooth. Basal segments of antennular peduncle bearing acute, irregular spines on anterior margin.

Chelipeds (Figs 1, 3) subequal, robust, dorsal surface somewhat convex, covered with interrupted scale-like ridges, granulated on ventral side. Merus with a large, serrate-edged tooth on anterior margin; carpus about 2.5 times as long as broad, with 4 rounded, serrated teeth on anterior margin, the proximal ones spine-tipped in some specimens; anterodistal edge with an additional blunt tooth; posterior margin with scale-like granules forming a row of 5 to 6 curved, upright spines, distally increasing in size. Manus moderately broad, posterior margin serrated but without spines, fringed with an irregular set of stiff and feathered setae; gape of fingers without distinct setation.

Walking legs (WL; Figs 1, 3) relatively robust, irregularly covered with scattered, simple and feathered setae of different size; merus of WL1 without spur-like spine on ventral, mid-distal margin; merus of WL1 and 2 with continuous transverse ridges; merus of WL3 without such ridges; merus of all WL with row of spines on anterior margin, distributed as follows: WL1 and 3: 5, WL2: 4-5. Merus of WL1 and WL2 with posterodistal spine; Dorsodistal edge of carpus in WL1 produced into a sharp spine. Propodus of all WL with 1 or 2 movable spines in addition to the terminal spine-triplet on posterior margin. Dactylus of all WL with 4 movable spines on posterior margin.

Coloration (Fig. 3). The carapace has a yellow-orange background with a white, reticulate pattern in the posterior half. The white markings on the gastric region fuse into an irregular, transversal stripe towards the epibranchial edges. The inverted figure of a butterfly is depicted by a broad, white, curved band connecting the hepatic margins on both sides, two semicircular white spots mesial to the orbits, and another backwardly curved white band behind the front. The reticulate pattern continues to the anterior part of the abdomen and the proximal parts of the walking legs, covering half to most of the merus. This pattern is then replaced by a dark purple band that increases in size, and is followed by a narrower white band bordering the articulation with the purple-colored carpus. Dactylus is also purple. White bands on both ends of the propodus give the walking legs a uniform, ring-like aspect. The chelipeds exhibit a similar ground color that becomes darker distally, with white marks forming irregular rows of round spots on carpus and manus.

##### Ecology.

The specimens examined were collected in depths between 7 and 23 m, on the outer reef slope, from *Halimeda* and dead *Pocillopora*. Further collections will probably confirm that *Petrolisthes paulayi* sp. n. inhabits other exposed coral environments of the tropical western Pacific.

##### Distribution.

The new species is known only from the Line and Society Islands in the Central Pacific.

##### Etymology.

The new species is named after Gustav Paulay for supporting this and other studies on Porcellanidae, and for entrusting us with the porcellanid collection of the Florida Museum of Natural History.

##### Remarks.

*Petrolisthes paulayi* sp. n. can be be easily distinguished from other *Petrolisthes* species of the Indo-West Pacific by its unique color pattern, and by the combination of the following characters on the carapace: two mesobranchial spines, two epibranchial spines and a conspicuous trilobate front. The later character is known only in *Petrolisthes elegans* Haig, 1981, which lacks mesobranchial spines, and only bears one epibranchial spine.

#### Petrolisthes aegyptiacus

Taxon classificationAnimaliaDecapodaPorcellanidae

Werding & Hiller, 2007

Fig. 4

Petrolisthes aegyptiacus Werding & Hiller, 2007: 5, fig. 4 (type locality: Egypt, Red Sea).

##### Material.

UF12962, 1 ovigerous female, Mascaréne Islands, La Réunion Island, Saint-Leu, Sec Jaune, rocky slope, basalt blocks, fore reef, under rocks, 10-19 m; UF13075, 1 male, La Réunion Island, Boucan Canot, Paine au Sucre, 10-15 m; UF33079, 1 male, 1 ovigerous female, Red Sea, Saudi Arabia, Thuwal, Al-Fahal reef, 1-37 m; UF36734, 1 specimen (identified from photograph), Red Sea, Saudi Arabia, offshore of Farasan Banks, Shib Radib, fore reef wall and barrier reef flat, 7-9 m; UF15472, 1 male, French Polynesia, Society Islands, Moorea Island, mid N coast, off Sheraton Hotel, outer reef slope, from within rubble; UF15474, 1 female (ovigerous), same data as UF15472.

##### Description.

**Figure 4.**
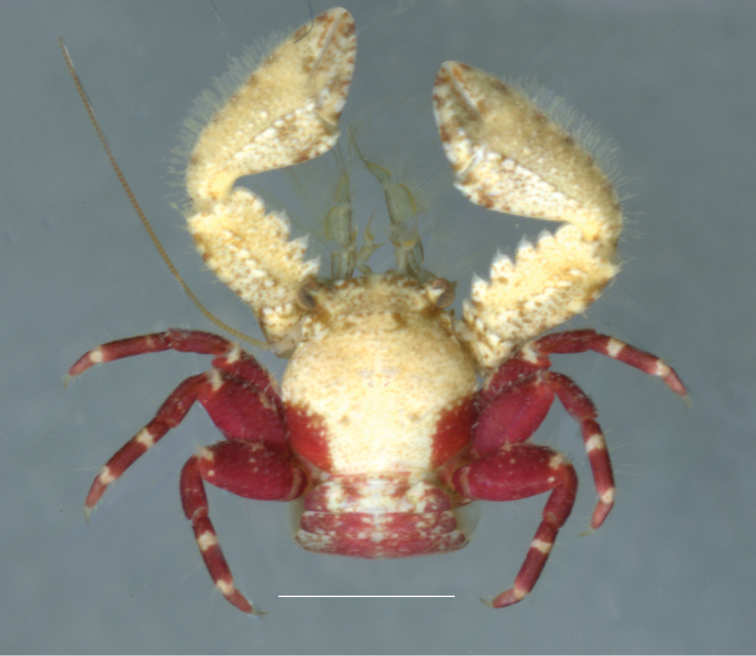
*Petrolisthes aegyptiacus* Werding & Hiller 2007, female, Red Sea, Egypt, Dahab. Scale bar: 3 mm.

Coloration. Fresh specimens from the Indian Ocean and the Central Pacific are white or beige on the anterior part of carapace and chelipeds (Fig. 4). The chelipeds may be entirely white, but usually show vivid red spots towards the fingertips. A row of small, purple spots may border the outer edge of carpus and manus. The front of the carapace may be fringed with some irregular, purple-brown spots, while a larger purple spot delimits the infra-ocular edge. The metabranchial regions bear a large, semi-lunar red blotch that extends towards the basal parts of the walking legs. The walking legs show a ring-like pattern. The surface of legs can be dark-purple or red with white marks, one of them on the distal margin of the merus, one at half distance of the propodus, and another near the articulation with the dactylus.

The red marks of the posterior part of the carapace extend to the lateral part of the segments of the abdomen that are visible from dorsal view. The median part of the first two or three segments of the abdomen is beige, interrupted by irregular red spots. The remaining posterior segments of the abdomen are entirely purplish. The whitish and reddish color, typical of *Petrolisthes aegyptiacus*, suggests a camouflaging strategy, as the color of the substrate inhabited by the species is usually spotted with red Foraminifera (*Homotrema* Hickson, 1911).

Distribution. Previously only known from the Red Sea and the Mascaréne Islands in the southwestern Indian Ocean, and the Society and Line Islands in the Central Pacific Ocean (for the occurrence in the Pacific, see discussion below).

## Discussion

*Petrolisthes paulayi* sp. n. was first found in the Line Islands, and subsequently in the Society Islands. The new species is probably confined to the Central and West Pacific, and seems to have a wide ecological range, as it occurs in shallow and deeper waters (7–23 m), and is adapted to different habitats, including corals and rocks. *Petrolisthes paulayi* sp. n. is morphologically most similar to *Petrolisthes heterochrous* because the two species bear an identical combination of carapace spines (Table 1). However, the new species is distinguishable from *Petrolisthes heterochrous* by its conspicuous, trilobate front. These two species can be distinguished from the other members of the mesobranchial-spine group by their lack of long, conspicuous setae on the carapace, typical of *Petrolisthes perdecorus*, and by the lack of a spur-like spine on the ventral, mid-distal margin of the merus of the first walking legs, specific of *Petrolisthes holthuisi* (see fig. 3 in Hiller and Werding 2010).

*Petrolisthes aegyptiacus* was originally considered a potential Red Sea endemic, based on few specimens collected in Quseir, Egypt, and Sanganeb Atoll, Sudan, and deposited in an old collection of the Museum für Naturkunde, Stuttgart, Germany (Werding and Hiller 2007). The species is here confirmed as a non-endemic of the Red Sea region, as the new material suggests that the species is also distributed in the southern Indian Ocean and the Central Pacific. However, preliminary comparisons of mitochondrial DNA sequences of specimens from the Indian Ocean and the Central Pacific (Hiller, unpublished data) suggest that specimens from the Line and Society Islands probably represent an undescribed species closely related to *Petrolisthes aegyptiacus*.

According to the present findings and those by Werding and Hiller (2007), no porcellanid species is endemic to the Red Sea. From the 18 species occurring in this sea, 12 extend their ranges into the West Pacific, and the rest into the southern Indian Ocean. These observations confirm those previously made by Lewinsohn (1969), who questioned the presence of endemic anomuran decapods in the Red Sea.

## Supplementary Material

XML Treatment for Petrolisthes paulayi

XML Treatment for Petrolisthes aegyptiacus
